# 

**Published:** 1886-05

**Authors:** 


### CONTENTS.

ORIGINAL COMMUNICATIONS—A Review of Antiseptic Wound Treatment, By A. C. Walker, M. D. Rockdale, Texas .......... 485
New Operation for Internal Hæmorrhoids, By J. M. Lewis, M. D., Mexia, Texas .......... 493
Foreign Bodies in the Rectum, By A. N. Perkins, M. D., Sabine Pass, Texas .......... 494
Vaginal Hysterotomy, By W. T. Collins, M. D. Grove, Texas .......... 495
SOCIETY NOTES—Galveston Medical Club; Differential Diagnosis, Diphtheria and Croup .......... 496
Summary of Proceedings Texas State Medical Association .......... 511
CONVENTION ITEMS— .......... 512
EDITORIAL—Dallas Meeting Texas State Medical Association .......... 515
St. Louis Meeting American Medical Association. 520
Who Shall Teach the Teachers? .......... 523
EDITORIAL NOTES—The Song of the Dying Swan .......... 524
Dangerous—Important Discussion—A Delusion and a Snare—"The Doctors that Meet in the Spring" .......... 526
Association American Medical Editors .......... 527
BIBLIOGRAPHY—The Story of Don Miff .......... 527
ADVERTISERS' NOTICES .......... 528–531

